# RNAi Knockdown of a Salivary Transcript Leading to Lethality in the Pea Aphid, *Acyrthosiphon pisum*

**DOI:** 10.1673/031.006.3801

**Published:** 2006-10-30

**Authors:** Navdeep S. Mutti, Yoonseong Park, John C. Reese, Gerald R. Reeck

**Affiliations:** ^1^ Department of Entomology, Kansas State University, Manhattan, Kansas 66506; ^2^ Department of Biochemistry, Kansas State University, Manhattan, Kansas 66506

**Keywords:** pea aphid, salivary gland, RNA interference, RNAi, small interfering RNA, siRNA

## Abstract

Injection of siRNA (small interfering RNA) into parthenogenetic adult pea aphids (*Acyrthosiphon pisum*) is shown here to lead to depletion of a target salivary gland transcript. The siRNA was generated from double stranded RNA that covered most of the open reading frame of the transcript, which we have called Coo2. The Coo2 transcript level decreases dramatically over a 3-day period after injection of siRNA. With a lag of 1 to 2 days, the siCoo2-RNA injected insects died, on average 8 days before the death of control insects injected with siRNA for green fluorescent protein. It appears, therefore, that siRNA injections into adults will be a useful tool in studying the roles of individual transcripts in aphid salivary glands and suggests that siCoo2-RNA injections can be a useful positive control in such studies.

## Introduction

Double-stranded RNA (dsRNA), when injected into or ingested by an organism or introduced into cells in culture, can specifically lower the level of the transcript of a target gene. This method, initially documented in *C.* *elegans* and named RNA interference, or RNAi, has become a very powerful tool to examine the role of individual genes (Fire et al. 1998; Zamore et al. 2000). Among insects, injections of RNAi in post-embryonic stages have been used successfully in the honeybee, *Apis mellifera* (Amdam et al. 2003), the moths *Hyalophora cecropia* (Bettencourt et al. 2002), *Spodoptera litura* (Rajagopal et al. 2002), *Bombyx mori* (Uhlirova et al. 2003) and *Manduca sexta.* (Levin et al. 2005), the mosquitoes, *Anopheles gambiae* (Osta et al. 2004) and *Aedes aegypti* (Attardo et al. 2003), the fruit fly, *Drosophila melanogaster* (Goto et al. 2003), a grasshopper, *Schistocerca americana* (Dong and Friedrich 2005), the red flour beetle *Tribolium castaneum* (Arkane et al. 2005a and 2005b, Tomoyasu et al. 2005,) and a termite, *Reticulitermes flavipes* (Zhou et al. 2006). Evidently, in some insect species, injected RNAi can move from the hemolymph into tissues or organs, where it then exerts its transcript-lowering effect, presumably by promoting degradation of the target mRNA. Extending this method to an aphid, and in particular, to the aphid salivary gland, is the objective of the work reported here.

Relatively long dsRNA is often used as an interfering RNA, but in two cases listed above (Levin et al. 2005 and Zhou et al. 2006) small interfering RNA (siRNA) was injected instead. siRNAs of 21-base pairs are highly effective in eliciting RNAi (Elbashir et al. 2001) and can be produced *in* *vitro* using the enzyme Dicer, a form of RNase III and the enzyme believed to produce siRNA *in* *vivo* (Bernstein et al. 2001).

Saliva is very important in the interaction between aphids and host plants. Proteins, including enzymes of aphid saliva have been postulated to play several roles, including the formation of a sheath around the stylets, the creation of an extracellular path by the stylet, overcoming plant defense and possibly stimulating plant defense in non-host plants (Miles 1999; Cherqui and Tjallingii 2000). As transcripts of potential interest are found in cDNA libraries of aphid salivary glands, (for instance, among the roughly 4500 pea aphid salivary expressed sequence tags (ESTs) recently deposited at NCBI as accession numbers DV747494 through DV752010), a method will be needed to examine the importance of transcripts of individual genes, and RNAi is a potentially powerful approach for doing so.

The most abundant cDNA from a salivary gland cDNA library prepared from the pea aphid, *Acrythosiphon pisum.* (Reeck et al., unpublished observations), was selected as the target transcript for this study. This cDNA was arbitrarily designated Coo2 (i.e., Cluster 2). This transcript was also found, infrequently, among whole-body and whole-insect pea aphid ESTs, where there are 7 occurrences in approximately 17,800 such ESTs deposited at the National Center for Biotechnology Information (Sabeter-Munoz et al. 2006). As a point of comparison, ESTs for cytochrome oxidase subunit-1, a widely distributed protein, occur over 160 times among the whole-body and whole-insect pea aphid ESTs. The translated nucleotide sequence of Coo2 does not match other sequences except in aphids, where there are matches to translated ESTs from *Aphis gossypii* and *Toxoptera citricida.* The entire open reading frame of the Coo2 transcript can be found at accession number CN763138. The encoded pea aphid protein includes a predicted signal peptide, and the protein is of entirely unknown function.

To test the efficacy of RNAi in aphids, siRNA coding for Coo2 was injected into adult aphids. It was found that injection of siRNA leads to knockdown of the Coo2 transcript level in salivary glands and results in a greatly reduced lifespan of the injected insects. These results provide the basis for the use of this technique in studies of other salivary transcripts and, possibly, transcripts in other organs.

## Materials and Methods

### Plants and aphids

Aphids were originally collected from alfalfa plants in the summer of 1999 by Dr. Marina Caillaud at Cornell University. Thereafter, the aphids were reared at KSU on fava beans (*Vicia* *fabae*) grown in pots (10 cm diameter) at room temperature under high intensity sodium lights with a L:D of 16:8. For the RNAi experiments, even-aged cohorts were established by collecting nymphs from young parthenogenetic females over a 24-h period. Cohorts formed in this way were maintained on plants for 7 days and then used for siRNA injections.

### Preparation of dsRNA and siRNA

PCR primers with T7 promoter sequences were used to prepare double-stranded RNA (Tomoyasu et al. 2005). For Coo2 RNA, the primers had the following sequences: 55′--TAA TAC GAC TCA CTA TAG GGA AGT TA--3′ and 5′--TAA TAC GAC TCA CTA TAG GGA AAC TT--3′ (forward and reverse, respectively). The two primers cover a region that extends from position 5 to position 637 in the open reading frame that, in its entirety, is 660 bases. Primers for green fluorescent protein RNA that were used in controls had the following sequences: 5′--TAA TAC GAC TCA CTA TAG GGC GAT GC--3′ and 5′--TAA TAC GAC TCA CTA TAG GGC GGA CT -- 3′ (forward and reverse, respectively). These cover a region of 520 bases in the open reading frame for the green fluorescent protein.

PCR products were gel purified using Qiaquick Gel Extraction Kit (Qiagen, www.qiagen.com).dsRNA was then made using Megascript RNAi Kit (Ambion, www.ambion.com) following the manufacturer's protocol. dsRNA was purified using phenol:choloform xtraction. siRNA was generated from dsRNA using the Dicer siRNA Generation Kit T5200002 (Genlantis, http://www.genlantis.com) and purified using siRNA purification columns of Genlantis. Products of Dicer digestion were checked for size (21 – 23 base pairs) on 15% acrylamide gels.

### siRNA injections

Glass needles (outer diameter of 1.0 mm, inner diameter of 0.50 mm; Sutter Instruments,http://www.sutter.com) were made using a micropipette puller (Model P-87, Sutter Instruments) at settings of: heat, 355; velocity, 50; time, 150. A PMI-200 pressure microinjector (Dagan, www.dagan.com) was used for siRNA injections. Aphids were held on their dorsa over a small hole in piece of plastic tubing (5 mm inner diameter) that was blocked at one end, held in place on a flat surface and connected at the other end to a small vacuum pump (Pro-Craft, Grobet USA, Carlstadt, NJ). Aphids were injected at the suture joining the ventral mesothorax and metathorax, at an angle of about 45 degrees, aimed toward the head of the aphid. We estimate that 5 nl of siRNA (10µg/µl) was injected into each aphid.

Leaves were cut from healthy, intact fava beans and put into a sterilized 2% agar (Fisher Scientific, www.fishersci.com) supplemented with 0.1% Miracle Grow fertilizer and 0.03% methyl 4-hydroxybenzoate (Sigma-Aldrich, www.sigmaaldrich.com) as a fungicide, essentially as described on the David Stern website (http://www.princeton.edu/%7Edstern/PlatesProtocol.htm) using Medicago leaves. About 7 ml of medium was placed in a Petri plate (100 × 15 mm) and one leaf was inserted into the agar as it cooled. One injected aphid was placed on each leaf. The plates were placed under GE Utility Shoplite with F48/25 watt/UTSL fluorescent lights, with 16:8 L:D, at a temperature of 23°C. Plates were checked several times a day for dead aphids, which were identified by lack of movement, being off the leaves and, after several hours, darkened coloration.

### Examining transcript levels by RT-PCR

Total RNA was isolated from individual pea aphid heads using TRI reagent (Molecular Research Center, Inc. www.mrcgene.com) following the procedure provided by the manufacturer. RNA was treated with DNase I (Ambion, www.ambion.com) following the company's instructions. AMV everse transcriptase was used with oligo-dT primers to synthesize single-stranded cDNA following the procedure in Technical Bulletin 099 of Promega. PCR was done using 5′--CCA GTG CGA TAG CGA TAA TTT ACA AC--3′ and 5′--CAC CTC TCT TAT GAT GAA CGC CAA C--3′ for C002 forward and reverse primers, respectively, giving a final product of 397 base pairs, and using 5′--CCG AAA AGC TGT CAT AAT GAA GAC C--3′ and 5′--GGT GAA ACC TTG TCT ACT GTT ACA TCT TG--3′ for ribosomal protein L27 forward and reverse, primers, respectively, giving a final product of 231 base pairs. The sequence of pea aphid L27 has accession number CN584974. Both primer pairs were used in each PCR, with L27 serving as an internal control. PCR was performed for one cycle at 95°C for 2 min followed by 26 cycles of: 95°C for 30 s, 54°C for 30 s and 72°C for 35 s. Primers were used at 0.3 µM and PCR master mix from Promega (www.Promega.com) was used in a final volume of 50 µl. PCR products were separated on 1% agarose gels prepared in 40 mM Tris-acetate (pH 8.3) and 1 mM EDTA. Ethidium bromide was added to a final concentration of 0.7 µg/ml before allowing the agarose to solidify. The gels were photographed under ultraviolet light and band intensities were obtained using SigmaScan's Pro5 image measurement software.

## Results and Discussion

**Figure 1.**
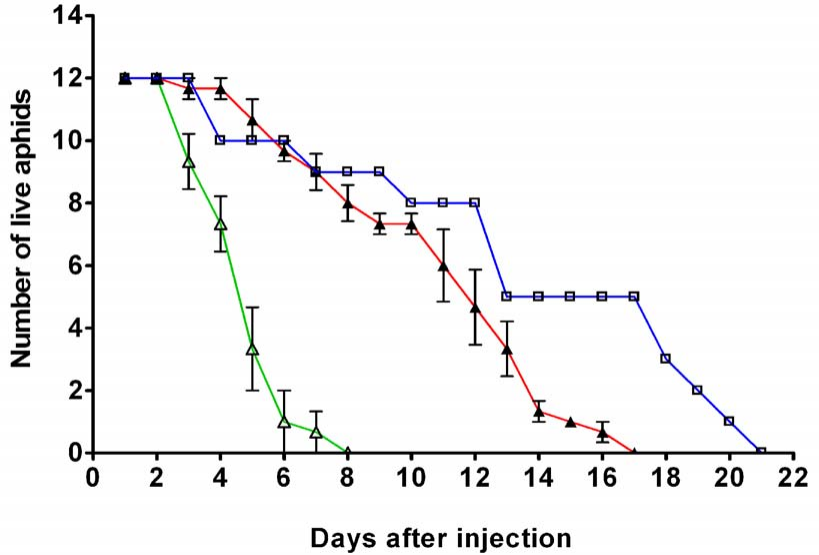
Survival of pea aphids after injection of siRNA. The graph shows the number of surviving aphids at daily intervals after injection. Green line and open triangles: injections with siCoo2-RNA. Red line and closed triangles: injections with siGFP-RNA (control). Blue line and open squares: uninjected insects. For the siRNA-injected insects, the data points are averages from three experiments, each of which began with 12 insects in the experimental and control groups. The bars depict standard errors.

Injection of siCoo2-RNA into adult parthenogenetic pea aphids led to greatly reduced life-span, as shown in Figure 1. Aphids injected with siCoo2-RNA died well before control aphids injected with green fluorescent protein si-RNA. Half of the aphids injected with siCoo2-RNA had died at 3 days after injection whereas 11 days was required for death of half of the aphids injected with control si-RNA. The survival of uninjected aphids was similar to that of aphids injected with control si-RNA, indicating that the small injection wound was tolerated by the aphids and that injection of control siRNA and buffer components did not have a toxic effect.

**Figure 2.**
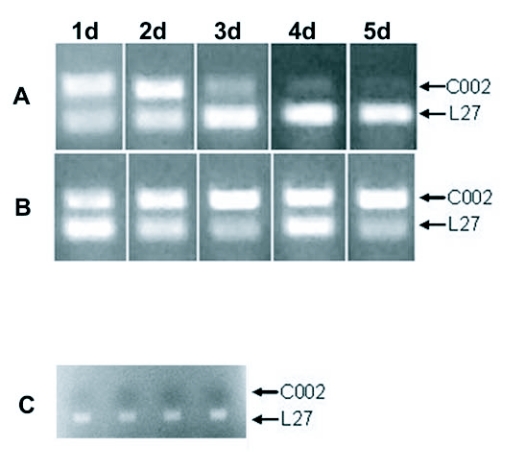
Knockdown of the Coo2 transcript after siRNA injections. Insects were injected either with siCoo2-RNA or siGFP-RNA. RNA from heads of injected insects was used in RT-PCR in which two primer-pairs were included, for Coo2 itself and for the transcript encoding ribosomal protein L27. The L27 PCR product serves as an internal control. The results shown (agarose gels after ethidium bromide staining) are of individual aphids at time points from 1 to 5 days after injection. Panel A: PCR products from reactions (26 cycles) with head RNA from siCoo2-RNA-injected insects. Panel B: PCR products from reactions (26 cycles) with head RNA from siGFP-RNA-injected insects. Panel C: PCR products from reactions (26 cycles) carried out on RNA extracted from heads from which salivary glands had been removed. Analysis of samples from 4 separate insects are shown.

RT-PCR was used to assess Coo2 transcript levels in RNA extracted from heads. The signal obtained by RT-PCR using head RNA as template is from transcripts in salivary glands, since using RNA from heads from which salivary glands had been removed by dissection gave no product in RT-PCR (Figure 2c). RT-PCR data from individual injected aphids are shown in Figure 2. Using the transcript for ribosomal protein L27 as internal control, we found that transcript levels from Coo2 dropped dramatically within 3 days after injection with siCoo2-RNA. On the other hand, in control si-RNA injected insects, Coo2 transcript levels, normalized to L27 transcript levels, did not change significantly.

**Figure 3.**
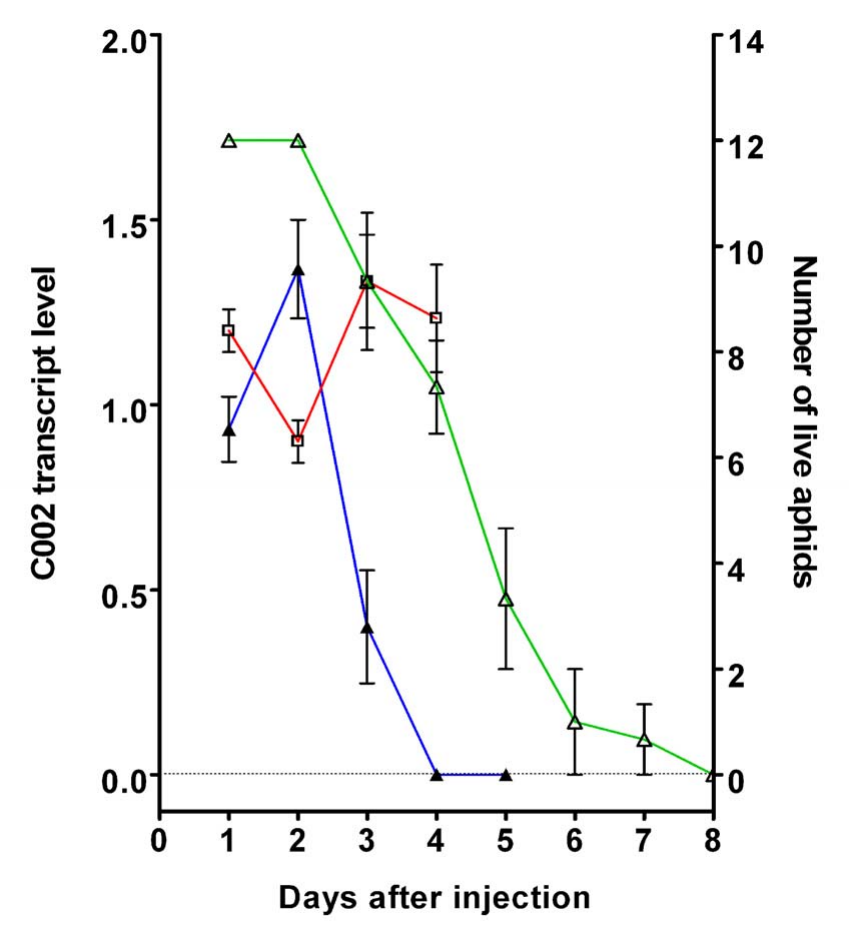
Timing of knockdown of Coo2 transcript after siRNA injection. Data from RT-PCR analysis of Coo2 transcript levels (normalized against L27 transcript levels) are plotted over a several day period after injection with either siCoo2-RNA (blue line, closed triangles) or siGFP-RNA (red line, open squares). Data are averages of normalized intensities from several individual insects at each time point and bars depict standard errors. The green line (open triangles) shows the survival of siCoo2-RNA-injected insects from Figure 1.

Semiquantitative data from such measurements are plotted in Figure 3 (open triangles), where, for purposes of comparison of the timing, the decline in the number of live insects (from Figure 1) is again shown. We note that the measurements of Coo2 transcript levels may somewhat overestimate the levels of the transcript and thus underestimate the timing of the knockdown, since in examining transcript levels only insects that were alive at each time point were used. Insects that had already died might well have had extensive knockdown of the Coo2 transcript.

These data demonstrate that siRNA injection into adult pea aphids can lower the transcript level of a target gene, Coo2, expressed in the salivary gland. The injections have a profound effect on lifespan, lowering the time to 50% survival from 11 days in control injections to about 3 days for injection of siCoo2-RNA. Several questions remain unanswered. Will the same procedure work for other salivary transcripts? There are no data at this time regarding this point, but we will soon undertake siRNA studies on other salivary transcripts. Will the same procedure work for lowering transcript levels in other organs? Again, there are no data on this point. Judging from results in other insects, it seems likely that gut (Rajagapol et al. 2002, Osta et al. 2004, Arakane et al. 2005), fat body (Attardo et al. 2003, Amdam et al. 2003) and hemocytes (Levin et al. 2005) would be sensitive to the effects of injected RNAi. Does siRNA enter aphid embryos developing within the injected adult female? We have not studied this systematically, but, informally, we have noticed that a significant fraction of nymphs from adults injected with siCoo2 RNA do die prematurely. This suggests the possibility that siRNA does indeed enter at least some embryos.

Unanswered by these experiments is the function of Coo2. We had no way of anticipating the profound effect of knockdown of the Coo2 transcript and in light of that effect, we will be investigating the function of Coo2 in ongoing experiments. One interesting piece of information that we have at this point is that siCoo2-RNA-injected aphids exhibit a peculiar behavior. Whereas uninjected aphids or aphids injected with siGFP-RNA stay quite still on the underside of the fava bean leaves, the siCoo2-RNA-injected aphids move around a good deal and do not stay confined to the underside of the leaves. Using electrical penetration graph methods we hope to understand the effect of Coo2 transcript knockdown on detailed aspects of the insects' feeding behavior.

## Abbreviations

RNAi: RNA interference
siRNA: small interfering RNA
dsRNA: double-stranded RNA
RT-PCR: reverse-transcriptase polymerase chain reaction
EST: expressed sequence tag

## References

[bibr01] Amdam GV Simoes ZLP Guidugli KR Norberg K Omholt SW. 2003 Disruption of vitellogenin gene function in adult honeybees by intra-abdominal injection of double-stranded RNA. *BMC Biotechnology* 3 1 12546706 10.1186/1472-6750-3-1 PMC149360

[bibr02] Arkane Y Muthukrishnan S Kramer KJ Specht CA Tomoyasu Y Lorenzen MD Kanost M Beeman RW 2005a The *Tribolium* chitin synthase genes *TcCHS1* and *TcCHS2* are specialized for synthesis of epidermal cuticle and midgut peritrophic matrix. *Insect Molecular Biology* 14 453 463 10.1111/j.1365-2583.2005.00576.x 16164601

[bibr03] Arakane Y Muthukrishnan S Beeman RW Kanost MR Kramer KJ 2005b *Laccase 2 is* the phenoloxidase gene required for beetle cuticle tanning. *Proceedings of the National Academy of Sciences USA.* 102 11337 11342 10.1073/pnas.0504982102 PMC1183588 16076951

[bibr04] Attardo GM Higgs S Klingler KA Vanlandingham DL Raikhel AS 2003 RNA interference-mediated knockdown of a GATA factor reveals a link to anautogeny in the mosquito *Aedes aegypti*. *Proceedings of the National Academy of Sciences USA* 100 13374 13379 10.1073/pnas.2235649100 PMC263821 14595016

[bibr05] Bernstein E Caudy AA Hammond SM Hannon GJ 2001 Role for a bidentate ribonuclease in the initiation step of RNA interference. *Nature* 409 363 366 11201747 10.1038/35053110

[bibr06] Bettencourt R Terenius O Paye I 2002 Hemolin gene silencing by ds-RNA injected into Cecropia pupae is lethal to next generation embryos. *Insect Molecular Biology* 11 267 271 12000646 10.1046/j.1365-2583.2002.00334.x

[bibr07] Cherqui A Tjallingii WF 2000 Salivary protein of aphids, a pilot study on identification, separation and immunolocalisation. *Journal of Insect Physiology* 46 1177 1186 10818245 10.1016/s0022-1910(00)00037-8

[bibr08] Dong Y Friedrich M 2005 Nymphal RNAi: systemic RNAi mediated gene knockdown in juvenile grasshopper. *BMC**Biotechnology* 5 25 10.1186/1472-6750-5-25 PMC1266053 16202143

[bibr09] Elbashir SM Harborth J Lendeckel W Yalcin A Weber K Tuschl T 2001 *Nature* 411 495 498 10.1038/35078107 11373684

[bibr10] Fire A Xu S Montgomery MK Kostas SA Driver SE Mello CC 1998 Potent and specific genetic interference by double-stranded RNA in Caenorhabditis elegans. *Nature* 391 806 811 9486653 10.1038/35888

[bibr11] Goto A Blandin S Royet J Reichhart J-M Levanshina EA 2003 Silencing of Toll pathway components by direct injection of double-strated RNA into *Drosophila* adult flies. *Nucleic Acids Research* 22 6619 6623 14602922 10.1093/nar/gkg852 PMC275548

[bibr12] Levin DM Breuer LN Zhuang S Anderson SA Nardi JB Kanost MR 2005 A hemocyte-specific integrin required for hemocytic encapsulation in the tobacco hornworm, *Manduca sexta*. *Insect Biochemistry and Molecular Biology* 35 369 80 15804572 10.1016/j.ibmb.2005.01.003

[bibr13] Miles PW 1999 Aphid saliva. *Biological Reviews of the Cambridge Philosophical Society* 74 41 85

[bibr14] Osta MA Christophides GK Kafatos FC 2004 Effects of mosquito genes on *Plasmodium* development. *Science* 303 2030 2032 15044804 10.1126/science.1091789

[bibr15] Rajagopal R Sivakumar S Agrawal N Malhotra P Bhatnagar RK 2002 Silencing of midgut aminopeptidase N of *Spodoptera litura* by double-stranded RNA establishes its role as *Bacillus thuringiensis* toxin receptor. *The Journal of Biological Chemistry* 277 46849 46851 12377776 10.1074/jbc.C200523200

[bibr16] Sabater-Munoz B Legeai F Rispe C Bonhomme J Dearden P Dossat C Duclert A Gauthier J-P Ducray DG Hunter W Dang P Kambhampati S Martinez-Torrez D Cortes T Moya A Nakabachi A Philippe C Prunier-Leterme N Rahbe Y Simon J-C Stern DL Wincker P Tagu D 2006 Large-scale gene discovery in the pea aphid *Acyrthosiphon pisum* (Hemiptera). *Genome Biology* 7 R21 16542494 10.1186/gb-2006-7-3-r21 PMC1557754

[bibr17] Tomoyasu Y Wheeler SR Denell RE 2005 Ultrabithorax is required for membranous wing identity in the beetle *Tribolium castaneum*. *Nature* 433 643 647 15703749 10.1038/nature03272

[bibr18] Uhlirova M Foy BD Beaty BJ Olson KE Riddiford LM Jindra M. 2003 Use of Sindbis virus-mediated RNA interference to demonstrate a conserved role of Broad-Complex in insect metamorphosis. *Proceedings of the National Academy of Sciences USA* 100 15607 15612 10.1073/pnas.2136837100 PMC307615 14668449

[bibr19] Zamore PD Tuschl T Sharp PA Bartel DP 2000 RNAi: Double-stranded RNA directs the ATP-dependent cleavage of mRNA at 21 to 23 nucleotide intervals. *Cell* 101 25 33 10778853 10.1016/S0092-8674(00)80620-0

[bibr20] Zhou X Oi FM Scharf ME 2006 Social exploitation of hexamerin: RNAi reveals a major caste-regulatory factor in termites. *Proceedings of the National Academy of Sciences USA* 103 4499 4504 10.1073/pnas.0508866103 PMC1450200 16537425

